# Trashing Property: Characteristics of Psychiatric Patients Who Engage in Domestic Property Damaging

**DOI:** 10.1007/s10597-019-00429-1

**Published:** 2019-06-10

**Authors:** Amber Postma, Sophie Bekmann, Johan M. Havenaar, Arjan W. Braam

**Affiliations:** 1 Department of Emergency Psychiatry, Altrecht Mental Health Care, Utrecht, The Netherlands; 2 Altrecht research section ‘Schroeder van der Kolk’, Utrecht, The Netherlands; 3 Department of Psychiatry, Canisius Wilhelmina Ziekenhuis, Nijmegen, The Netherlands; 4 Crisisdienst Utrecht, Altrecht, Lange Nieuwstraat 119, 3512 PG Utrecht, The Netherlands

**Keywords:** Domestic property damage, Emergency Psychiatric Services, Crisis resolution, Psychiatric, Aggression

## Abstract

This descriptive record-based study included 75 patients who had engaged in domestic property damaging (DPD) and needed assessment by an urban emergency psychiatric service team in The Netherlands. The DPD patients were compared to 1145 other patients referred because of aggression, suicidality or other reasons. DPD patients were more often diagnosed with a psychotic disorder or a manic episode, had more often a migration background, were less often diagnosed with depression, and had lower GAF scores. There were no differences with respect to personality disorders or substance use. DPD patients were two to six times more likely to be (mostly involuntarily) admitted to a psychiatric department (64%), than the other patient groups (aggression 45%, suicidality 21%, other referral reasons 37%). The findings indicate that DPD patients represent an exclusive group who possibly have more intercultural and communication disadvantages due to less cultural acceptance or lack of knowledge about mental healthcare in the Netherlands.

## Introduction

The connection between violence and mental illness is often a topic of debate (Friedman 2006). Frequently, the question arises whether patients with mental illness pose an increased risk of violence. Much of the current literature on violent behaviour pays particular attention to deliberate self-harm and physical violence towards others. A different kind of violent behaviour is domestic property damaging (DPD). DPD can consist, for example, of breaking crockery, throwing furniture through the window or setting a fire to one’s home. When people engage in DPD and when their mental state appears to be disordered, the police is likely to request assessment by the Emergency Psychiatric Services (EPS) (Johnson and Thornicroft 2008). DPD can be assumed to represent a prototypical presentation to the EPS. The phrase ‘DPD patients’ is defined by the particular type of violence: DPD patients use confined violence towards household property but not against others or themselves. Nevertheless, these patients engage in fierce violence with sometimes serious material consequences as well as profound disapproval by others such as neighbours or house owners. One may question whether DPD either represents an extraordinary, disproportionate way of communicating distress and thus a cry for help or just an extreme form of aggressive behaviour, where DPD may represent a distinguishable subgroup among patients with internalising and externalizing aggression. Although aggression receives considerable attention in the psychiatric research literature, DPD has rarely been described. Describing the characteristics of DPD patients could help to identify these patients and clarify their particular behaviour and so to facilitate targeted mental healthcare interventions.

The literature describes different characteristics of patients who engage in violence. Several risk factors have been identified that play a role in aggressive behaviour (Freedman et al. 2007; Siever 2008). Environmental factors include familial factors, such as having observed or experienced aggression as a child or adolescent, as well as cultural and socioeconomic factors conducive to aggression. Furthermore, violent behaviour has been associated with psychiatric disorders and particularly in patients with antisocial and borderline personality disorders (Yu et al. 2012; de Barros and de Pádua Serafim 2008; Freedman et al. 2007; Siever 2008). Another characteristic of patients who engage in violence could be the migrant status (Engbersen et al. 2007; Junger-Tas 1997). The association between the migrant status and (violent) crimes has been a subject of recurrent public discussions (Engbersen et al. 2007; Moehling and Piehl 2009). In the Netherlands the highest crime rates are found among male migrants who are classified as non-Western (Engbersen et al. 2007). Likewise, young non-Western immigrants report more often than young natives to have committed violent offenses and offenses against property. Nonetheless, the question on crime and migrant status could also be based on ethnic stereotyping, cultural racism and subsequent increased surveillance and reporting of crime (Siebers and Dennissen 2014; Junger-Tas 1997).

Many studies have illustrated the association between violence and multiple factors (Freedman et al. 2007; Siever 2008). Substance abuse, environmental stressors, a history of violence, hostile behaviour, non-adherence with psychological therapies and poor impulse control, non-adherence with medication, recent substance misuse and substance abuse comorbidity are factors described as associated with violent behaviour (Witt et al. 2013). Engbersen et al. (2007) concluded from their study among 34,653 subjects diagnosed with a mental illness that severe mental illness alone did *not* predict future violence. Moreover, Fazal et al. (2009) conclude that most of the excess risks appears to be mediated by comorbid substance abuse. The risk among substance abuse patients with comorbid psychotic illness is similar to the risk without psychotic illness. Although the comparison of the different types of violence was beyond the scope of these studies, a pilot study by Ben-Zeev et al. (2017) found an association among hospitalised patients between damaging property, physical aggression and symptoms of substance withdrawal.

Likewise, the literature is inconclusive on the management of psychiatric patients after engaging in violent behaviour. In a study conducted on reasons of referral to the EPS in Utrecht, The Netherlands, 29% of the patients with the referral ‘aggressive behaviour’ were admitted involuntarily (Hoek and Braam 2017). Parts of these data are used for the current study as well. The rate of involuntary admissions in psychiatric hospitals is known to show large variations across sites (van der Post et al. 2009; Gandré et al. 2017; Zhou et al. 2015; Zinkler and Priebe 2002). For example, in Utrecht, the mean prevalence of involuntary admissions has been described to be 4.4 per 10,000 inhabitants per year whereas in Amsterdam, the prevalence of emergency involuntary admissions amounted to 8.6 per 10,000 inhabitants (Braam et al. 2016; van der Post et al. 2009). Although many studies describe the prevalence of involuntary admission and the characteristics of these patients, these studies do not make a distinction in the type of violence (van der Post et al. 2009; Braam et al. 2016; Hoek and Braam 2017).

The current descriptive medical record study aims to describe (a) the prevalence of referrals to the EPS of patients engaging in DPD, (b) the association with different factors possibly playing a role in violent behaviour including possible problems in communication (due to less knowledge about care facilities, intoxicated states, interpersonal communication problems), and (c) which management options are mostly decided to with respect to DPD patients. The study was conducted at the EPS in Utrecht, the Netherlands, operating in the city of Utrecht and its surrounding suburban municipalities.

## Methods

### Study Design and Participants

The current study is a descriptive medical record study which took place at the EPS of Altrecht Mental Health Care, research section ‘Schroeder van der Kolk’. The EPS of Altrecht operates in the city of Utrecht, with approximately 340,000 inhabitants, and about 200,000 inhabitants in the surrounding suburban region. The study was approved by the Scientific Committee of Altrecht Mental Health Care (2010-14/oz1007).

The patients were included in six separate assessment periods between 2009 and 2016 which were (1) 25-03-2009 up to including 24-04-2009; (2) 18-10-2010 up to including 22-11-2010; (3) 10-09-2012 up to including 21-10-2012; (4) 24-04-2014 up to including 07-06-2014; (5) 10-01-2015 up to including 15-02-2015; (6) 19-09-2016 up to including 25-10-2016. In these assessments periods the records of DPD patients and of EPS comparison patients were collected consecutively. A part of the subsample of the DPD patients was collected outside the assessment periods and was collected in a consecutive series from 22-10-2006 up to 12-12-2010 in the daily reports at the EPS of Utrecht. Thus, the DPD patients are partly identified by the above assessment periods and partly aside from the assessment periods.

The DPD subsample consists of patients who were referred to the EPS with the reason of DPD (A). The comparison patients were divided into three groups based on reason of referral: patients with aggression not restricted to DPD (B) patients with suicidal behaviour not restricted to DPD (C) and patients with the remaining referral reasons not restricted to DPD (D). Aggression as reason of referral has been chosen as a comparison group to verify whether DPD patients represent a type of aggression that needs a different understanding and perhaps a different clinical approach. Suicidality as a reason of referral and the remaining reasons of referral are chosen to determine the difference between DPD patients and the main EPS population. A total of 1220 patients was included in the study in which 75 of the cases engaged in DPD (A), 156 patients were included in the aggression group (B), 543 patients were included in the suicidal group (C), and 446 patients were included in the group with remaining reasons of referral (D). Patients who engage in other types of violent behaviour are referred to with the term ‘aggression’. The ‘remaining referrals’ groups (D) included intoxicated states (not restricted to DPD, aggression or suicidality), cognitive problems, and depression and anxiety states (such as related to post traumatic stress, panic disorder or relational problems, without the restriction to suicidality).

### Characteristics and Variables

The key information for the current study was derived from the Altrecht EPS patient records, utilizing a coding form for each record. This form was filled in by medical students or staff members of the EPS. The majority of the forms were filled out completely. About three quarters of the forms were double checked by a senior psychiatrist at the EPS (ABr). The form included the following characteristics: age, gender, the place of residence, referring agency, referral reason, location of administration, current legal status, psychiatric medical history, substance use, cultural background, working diagnosis of the acute mental state, any known previous diagnosis of a personality disorder, GAF score, and the management after the intake. Not all characteristics of the form were included in the study. With respect to the current legal status patients were divided into those who were under Dutch Mental Health Act and patients who were not (van Tilburg et al. 2008). Substance use, evaluated by a social worker or psychiatrist at the EPS, has been divided into substance abuse on the one hand, and no use, denial or non-registration, on the other hand. The migrant status, as is routinely recorded in new patients at registration, was defined by either place of birth of the parents outside the Netherlands, and in some cases, where this was not recorded, by a non-Dutch family name in combination with the description of cultural background as aspect of appearance in the mental status examination in the psychiatric examination. The main diagnoses (as established at the time of the crisis intervention) were classified into four groups: psychosis, mania (bipolar disorder), depression and other diagnoses (cognitive disorder, anxiety disorder, adjustment problems, impulse control disorder, relation problems, attention deficit hyperactivity disorder). The Global Assessment of Functioning (GAF) scale is a scale between 0 and 100, which considers the psychological, social and occupational functioning on a hypothetical continuum of mental health-illness (American Psychiatric Association 1994). The management of the patients after assessment by the EPS was categorised into ‘admission’, with subdivisions ‘voluntary’ and ‘involuntary’ admission, and a composite category of other interventions: e.g. appointment with the regular treatment team of the patient (*within 1 or 2* *days*), new appointment with the EPS (*within 1 or 2* *days*), back-referral to the general practitioner’. In The Netherlands, with short geographical distances to care facilities, there is an increasing reliance on high frequent, acute out-patient care (such as Intensive Home Treatment; Cornelis et al. 2018).

### Statistical Analysis

The characteristics derived from the medical records were compared to identify the main differences between de DPD patients and the three other groups taken as one and separately. For one variable, the GAF-score, bivariate associations were analysed using T-tests for comparing mean scores and comparing ratios for bivariate associations were analysed, computing mean scores for each of the four groups (A, B, C, D), testing for significant differences using T-tests. For all other associations, multivariate logistic regression models were employed, including demographic, diagnostic and care variables, computing Odds ratios and 95% confidence intervals. The main types of management as applied at the EPS were compared within these groups by using Odds ratios. There are no known conflicts of interest for any of the authors. All authors certify their responsibility for the manuscript.

## Results

### Incidence and Forms of DPD

Among all presentations to the EPS, the 30-days incidence of presentations with DPD amounted to 1.2%. On average 151 patients were referred to the EPS in 30 days. This is tantamount to about 1 to 2 DPD patients being referred to the EPS every 30 days in the care region with about 540,000 inhabitants. With respect to how the DPD manifested itself the records provided some additional information on the type of DPD. Table 1 illustrates five subclasses of DPD and differences between the groups.

**Table 1:** Frequencies and admission rate with respect to the types of domestic property damaging which was apparent in the records of the Emergency Psychiatric Services in Utrecht, The Netherlands

Type of domestic property damaging	Explanation	Frequency	Admission
		% (n)	% (n)
Crockery	Only having destroyed some crockery (e.g. glassware, paintings)	45 (34)	47 (16)
Trashing everything	Dutch translation: ‘kort en klein geslagen’ which was often used as a general description for patients who completely destroyed their furniture/household	24 (18)	78 (14)
Throwing things outside	Having thrown household through the window or down the stairs at the apartment complex	17 (13)	92 (12)
Trashing and throwing outside	Having completely destroyed house/household and having thrown household through the window or down the stairs at the apartment complex	4 (3)	100 (3)
Fire/water damage	For example: having flooded the apartment or started a fire in the apartment	9 (7)	43 (3)

### Characteristics of DPD Patients in Comparison with Other Groups at the EPS

Table 2 summarizes the demographic, diagnostic and care characteristics of the DPD patients (A) and the other groups (B, C, D). The results of the multivariate analyses (GAF scores excluded, because the estimation of the GAF score would heavily depend on DPD) are shown in the right-hand panel of Table 2. In comparison with the aggression group (B), the DPD patients suffered more often from mania. In comparison to the suicidality group (C), the DPD patients were significantly more often male, migrant, suffering from psychosis or mania and were less often diagnosed with depression. In comparison with the other referrals (D), the DPD group was significantly more often male. With respect to age there is no significant difference between the groups.

**Table 2:** Characteristics of subsamples referred to the emergency psychiatric services, because of domestic property damaging (DPD = A), aggression (B), suicidality (C), and ‘other’ referral reasons (D); rates and comparisons to the DPD group using multivariate logistic regression analyses (significant associations are marked in bold)

	A (n = 75)	B (n = 156)	C (n = 543)	D (n = 446)	A vs B, C, D	A vs B	A vs C	A vs D
	% (n)	% (n)	% (n)	% (n)	OR (CI)	OR (CI)	OR (CI)	OR (CI)
Gender (male)	**76 (57)**	78 (122)	**44 (238)**	**53 (236)**	**2.31 (1.24–4.33)**	0.95 (0.44–2.04)	**4.10 (2.01–8.36)**	**2.54 (1.31–4.92)**
Age	38.9y	41.5y	38.7y	42.5y	1.01 (0.99**–**1.02)	1.00 (0.97**–**1.02)	1.02 (1.00**–**1.04)	1.0 (0.98**–**1.02)
Psychosis	**47 (33)**	34 (53)	**7 (40)**	33 (146)	**2.75 (1.39–5.43)**	2.00 (0.89–4.48)	**8.61 (3.91–18.98)**	1.40 (0.68**–**2.87)
Mania	**14 (10)**	**8 (13)**	**2 (13)**	10 (42)	**3.28 (1.35–7.96)**	**5.78 (1.74–19.2)**	**13.66 (4.44–42.04)**	1.65 (0.65**–**4.20)
Depression	**3 (2)**	3 (4)	**34 (184)**	14 (61)	**0.21 (0.05–0.93)**	1.58 (0.26**–**9.75)	**0.13 (0.03–0.60)**	0.28 (0.06**–**1.29)
Other diagnoses^a^	36 (25)	55 (85)	56 (304)	43 (191)	1^a^	1^a^	1^a^	1^a^
Cultural background (migrant)	**43 (32)**	36 (47)	**20 (101)**	31 (115)	**1.80 (1.03–3.15)**	1.15 (0.57**–**2.33)	**3.59 (1.78–7.22)**	1.66 (0.92**–**2.98)
Substance use %	46 (34)	48 (66)	37 (186)	40 (151)	0.84 (0.47**–**1.52)	1.08 (0.52**–**2.24)	1.07 (0.53**–**2.16)	0.75 (0.39**–**1.43)
In mental healthcare	68 (50)	67 (92)	58 (289)	54 (200)	0.79 (0.44**–**1.35)	1.29 (0.67**–**2.48)	0.83 (0.43**–**1.64)	0.63 (0.35**–0**.1.14)
Already under Mental Health Act	7 (5)	8 (11)	3 (14)	9 (33)	0.76 (0.27**–**2.13)	0.71 (0.21**–**2.42)	1.17 (0.28**–**4.87)	0.68 (0.24**–**1.97)
Personality disorder	23 (16)	36 (56)	43 (185)	21 (91)	0.77 (0.41**–**1.46)	0.55 (0.26**–**1.14)	0.71 (0.34**–**1.48)	1.11 (0.56**–**2.18)
GAF score	37.6	42.1	46.1	40.5				

GAF scores are not included in the multivariate model, as determining the score heavily depends on classifying a situation as ‘DPD’ (tautology)

^a^Reference group

The GAF scores (analyzed separately) were significantly lower in the DPD group (37.6) compared with the other groups combined (T = 4.1, P = 0.000), the aggression group (T = − 2.4, P = 0.020) and the suicidality group (T = − 6.3, P = 0.000). There was no significant difference with the ‘other referrals group’ (T = − 1.8, P = 0.066).

### Management of DPD Patients

Table 3 pertains to the management within each group and shows differences between the rates of deciding to involuntary admission, voluntary admission and other interventions. Within the DPD group, the admission rates amounted to 64%:43% were involuntarily admitted and 21% voluntarily. The DPD patients were two (as compared to patients referred because of aggression) to six times (as compared to patients referred because of suicidality) more often admitted to a psychiatric hospital than the other patient groups.

**Table 3:** Association between policies for patients who engaged in domestic property damaging (DPD) compared with the different groups

	Involuntary admission	Voluntary admission	Other policies^a^	Admission vs other policies for DPD patients compared to other groups	Involuntary vs voluntary admission for DPD patients compared to other groups
	% (n)	% (n)	% (n)	OR (95% CI)	OR (95% CI)
DPD	43 (32)	21 (16)	36 (26)	–	–
Overall (B, C, D)	16 (175)	15 (170)	70 (782)	**4.03 (2.47–6.57)**	**1.94 (1.03–3.67)**
Aggression (B)	33 (52)	12 (19)	55 (85)	**2.13 (1.21–3.75)**	0.73 (0.33–1.62)
Suicidality (C)	8 (41)	13 (72)	79 (423)	**6.66 (3.98–11.14)**	**3.51 (1.72–7.16)**
Other referrals (D)	19 (82)	18 (79)	63 (274)	**3.03 (1.82–5.04)**	1.93 (0.98–3.78)

The values are highlighted in bold so the reader can easily comprehend the results

OR = DPD (A) vs the overall group (B, C, D); vs the aggression group (B); vs the suicidality group (C); and vs the other referrals group (D)

^a^Composite category including management options such as referral to the treatment team of the patient, new appointment with the EPS, or back-referral to the general practitioner

## Discussion

### Main Results

The aim of the current exploratory study was to examine characteristics of patients who damage their own household property and who were assessed by the EPS in Utrecht, The Netherlands. We compared the DPD patients to several other groups by reason of referral. Compared to patients referred because of suicidality, DPD patients were more often male, migrant, suffering from psychosis or mania and less frequently depressed. In comparison with patients referred because of aggression, the DPD patients were more frequently diagnosed with mania. With respect to age, substance use and personality disorder, no significant differences were observed. The DPD group had significantly lower scores on the GAF scale and had a higher chance of being hospitalised involuntarily and voluntarily (up to 64%) as compared to the other groups.

### Comparison with Previous Studies

Many studies have illustrated the association between violence and various factors, such as substance use, personality disorder and environmental stressors (Freedman et al. 2007; Siever 2008; Elbogen and Johnson 2009; Fazal et al. 2009; Witt et al. 2013). The results of the current study did not identify substance use as a risk factor for DPD. However, the EPS patients in Utrecht were not tested for substance use, and patients who were scaled into the ‘no substance use’ category could still have used substances. In addition, DPD patients were possibly less capable of providing correct information about their substance use. Possibly, substance abuse remains a substantial risk factor for all types of violence, including DPD, irrespective of the presence of a psychotic disorder (Fazal et al. 2009).

The results illustrate that the DPD group had the largest percentage of patients with a migrant background. Since several decades, the Netherlands have received many migrants, labour migrants, as well as refugees, from a wide range of countries (Statistics Netherlands 2013). Patients with a migration background are likely to have a disadvantage with respect to the Dutch language. Research points out that only 3% of people with a language disadvantage in the Netherlands takes an interpreter to the doctor (Lamkaddem et al. 2013). A study on equal access of mental healthcare services in the Netherlands illustrates that for the non-Dutch-speaking migrants, utilisation of the mental healthcare only reached about half the level of the Dutch citizens (Koopmans et al. 2013). A language disadvantage and cultural habits can influence (and discourage) help-seeking behaviour of migrants born in the Netherlands as well. Due to socio-cultural and personal situations, a migrant can question the additional value of the available mental healthcare in the country of residence which can lead to a substantial patient-delay (Straβmayr et al. 2012). Another Dutch study on compulsory admissions and clinical presentation among immigrants at the EPS in Rotterdam found that first- and second-generation immigrants from non-Western countries to be at a higher risk of contact with EPS than members of the native Dutch population (Mulder et al. 2006). Mulder et al. explained the association between the non-Western ethnicity and compulsory admission by the greater severity of psychiatric symptoms, greater level of threat, lack of treatment motivation and lower level of functioning. Moreover, Selten et al. suggests in a study on social defeat as a hypothesis of schizophrenia that migration and childhood trauma are the strongest association for the increased risk on schizophrenia (Selten et al. 2013).

Nonetheless, Morgan et al. describe two American studies that found no association between ethnicity and compulsory admission but found an association between compulsory admission and living alone or living far away from family which Post van der et al. also concluded (Morgan et al. 2004). Furthermore, Morgan et al. suggests that because of the stigma attached to mental illness among some communities, immigrants do not follow the usual pathway to mental healthcare, have a patient delay or do not seek help at all. Less access to mental healthcare or inadequate utilisation of the mental healthcare can emerge from a culturally based lack of acceptance, prejudice and lack of knowledge about access and possibilities of mental healthcare. Therefore, it can be an obstacle to seek help or start the suggested therapy. Consequently, a high chance arises that these patients do not get the proper healthcare which ultimately may result in an escalation such as DPD.

Way et al. concluded that suicide potential, danger to others, and symptom severity were the best predictors of an admission in a healthcare center (Way et al. 2001). However, other studies show low rates of admittance of patients with suicidality which is more in line with findings of the current study (Mulder et al. 2005; George et al. 2002). Apparently, there is a different approach to the referral for suicidality between countries (Mulder et al. 2005). Another argument is that some families have the preference for the patient not to be hospitalized (Mulder et al. 2005). Making a distinction between personality disorder and suicidality can reflect on the suicidality referral rates. Breslow et al. suggest that suicidal patients are more likely to be discharged because of the strong association with personality disorder (Breslow et al. 1993). Patients with personality disorders are easily overwhelmed which can lead to suicidality. The current study made a distinction between suicidality and other referrals (including personality disorders). Nonetheless, personality disorders may have been underdiagnosed, as such diagnoses usually take a more lengthy and in-depth assessment than is usually available in psychiatric emergency conditions, where, more often than not, other clinical priorities may prevail. These arguments may to some extent explain the difference in admittance rates of patients with suicidality among these studies.

Several studies suggest that personality disorders, especially borderline and antisocial personality disorders, are associated with a higher chance of engaging in impulsive or violent behaviour (Yu et al. 2012; de Barros and de Pádua Serafim 2008; Freedman et al. 2007; Siever 2008). The current findings do not support this assumption with respect to DPD patients. The results do not reveal a significant association with personality disorder and DPD or other forms of violence. It seems not unlikely that personality disorders may have been underdiagnosed, as suggested before. Personality disorder may therefore remain unrecognised as a major underlying reason for acute psychiatric presentations such as psychosis or mania. This may explain the current findings.

The literature is inconclusive on the management of psychiatric patients after engaging in violent behaviour. An earlier study describes that 29%, of those who were referred to the EPS of Utrecht because of aggression, were admitted involuntarily (Hoek and Braam 2017). However, prevalence figures of involuntary admission show considerable variation between countries (van der Post et al. 2009; Gandré et al. 2017; Zhou et al. 2015). The current study has found a 43.2% involuntary admission within the DPD group, and within the overall comparison (B, C, D), a 15.5% involuntary admission. Altogether, the DPD patients have a significant higher chance of admission (OR = 4.03, CI 2.47–6.57) in comparison to the overall group. Some of the characteristics, described in literature, of patients who are admitted involuntarily are similar to the DPD group such as male gender and psychotic disorders (van der Post et al. 2009, 2012; Gandré et al. 2017; Zhou et al. 2015). Some additional analyses even showed that the rate of admission the patients in the ‘trashing everything’ and ‘throwing things outside’ groups even amounted to 85%. Admission could be considered as a plausible, temporary part of management, and preferred to example Intensive Home Treatment (Johnson and Thornicroft 2008), because the crisis apparently emerged in the domestic context.

### Limitations

Several limitations of the current, exploratory study need consideration. First, the reason for referral was not always entirely obvious: there is a possibility that patients fitted in more than one group. Consequently, it frequently depended on the opinion of the investigators, staff members and the senior psychiatrist, which category was chosen. In addition, medical records occasionally contain too concise or inaccurate information leading to missing values in the study. Inaccurate and possibly unreliable information with respect to substance use and abuse has already been mentioned. In addition, the assessment of migrant status could be inaccurate as well where in cases, when parental place of birth was not registered, it was determined on the basis of a non-Dutch family name in combination with the description of cultural background in the mental status examination, which was generally derived from earlier information in the patient’s file. However, in The Netherlands, the majority of non-Western migrants originating from four well acknowledged regions: Morocco, Turkey, The Dutch Caribbean Islands and the previous colony of Surinam (Centraal Bureau voor de Statistiek 2018).

Furthermore, it is questionable whether the term ‘DPD’ would only be applicable to psychopathological states. People can also deliberately damage their belongings, or belongings of the owners of their accommodation, for purposes such as expecting a renovation or hurting someone’s feelings. The intention of the DPD is far from evident. Therefore, the term, as meant in the current study, should be ‘DPD during psychopathological states’.

## Conclusion

Patients who engage in DPD show signs of acute distress and loss of any adequate problem-solving behaviour, except that they do not use aggression towards other people. DPD patients suffer more often from psychosis and mania, are more likely to be migrant and do not necessarily suffer from personality disorders and (as far as the data allow to reveal) substance use disorders. This paper provides some preliminary evidence that, at least in the Netherlands, DPD represents a distinguishable subgroup among patients with externalizing aggression. These patients may have special needs to attend to during the care process e.g. they may have more intercultural and communication disadvantages due to less knowledge about care facilities, cultural habits and lack of understanding and prejudice amongst the public resulting in violent escalations such as DPD.

Clinical consequences of the current study pertain to more recognition for this crisis phenomenon as well as to future research. For future research, we suggest focussing on how DPD patients perceive their need of care as well as their obstacles to communicate about their needs with relevant others. DPD patients might be interviewed and invited to reflect on their actions and on their intentions at the time of the DPD. Deepening our understanding of this behaviour will be a next step in our future research. Furthermore, it would be relevant to know whether DPD patients show other types of aggressive behaviour over time, e.g. in previous episodes. The ideas of DPD patients about the origins (lack of access to care) and consequences (stigmatisation, regret, financial problems) of their behaviour could be further assessed. An example of prevention may include formulating a crisis plan with relatives and mental healthcare professionals generating a better or earlier access to mental health care in future crisis situations. Besides these suggestions, further research should also prevent any xenophobic motivations towards non-natives and the possible risk for the society suggested in these results. As suggested in this study migrants might not be able to follow the usual pathway in the mental healthcare. This is a responsibility of healthcare authorities to make this pathway more accessible and prevent escalated situations like DPD.
